# Supplementary material for: The causal relationship between the human gut microbiota and pyogenic arthritis: a Mendelian randomization study
Source: Front Cell Infect Microbiol. 2024 Nov 26;14:1452480. doi: 10.3389/fcimb.2024.1452480 (PMC11629706; doi:10.3389/fcimb.2024.1452480)
Supplement: Supplementary file 1 [file DataSheet1.zip › mendelian test/ebi-a-GCST90027507.csv_forest.pdf]

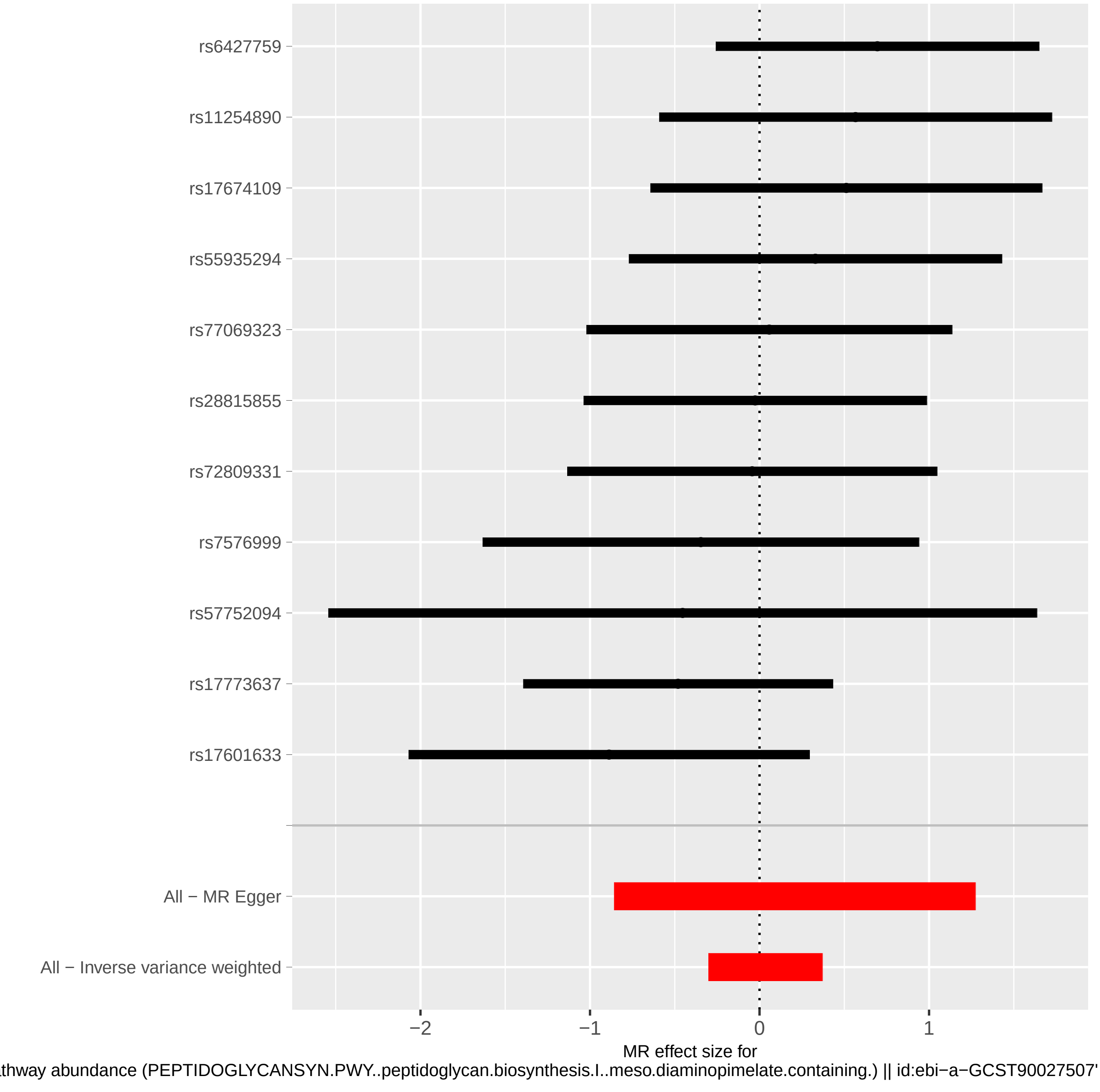
rs6427759
rs11254890
rs17674109
rs55935294
rs77069323
rs28815855
rs72809331
rs7576999
rs57752094
rs17773637
rs17601633
All − MR Egger
All − Inverse variance weighted
−2
−1
0
1
MR effect size for
thway abundance (PEPTIDOGLYCANSYN.PWY..peptidoglycan.biosynthesis.I..meso.diaminopimelate.containing.) || id:ebi−a−GCST90027507'
